# Production and purification of endogenously modified tRNA-derived small RNAs

**DOI:** 10.1080/15476286.2020.1733798

**Published:** 2020-03-05

**Authors:** Aleksej Drino, Vera Oberbauer, Conor Troger, Eva Janisiw, Dorothea Anrather, Markus Hartl, Steffen Kaiser, Stefanie Kellner, Matthias R. Schaefer

**Affiliations:** a Division of Cell and Developmental Biology, Center for Anatomy and Cell Biology, Medical University Vienna, Vienna, Austria; b Mass Spectrometry Facility, Max Perutz Laboratories (MPL), Vienna Biocenter (VBC), Vienna, Austria; c Department of Chemistry, LMU Munich, Munich, Germany

**Keywords:** tRNA, tRNA fragments, RNA modifications, stress

## Abstract

During particular stress conditions, transfer RNAs (tRNAs) become substrates of stress-induced endonucleases, resulting in the production of distinct tRNA-derived small RNAs (tsRNAs). These small RNAs have been implicated in a wide range of biological processes, but how isoacceptor and even isodecoder-specific tsRNAs act at the molecular level is still poorly understood. Importantly, stress-induced tRNA cleavage affects only a few tRNAs of a given isoacceptor or isodecoder, raising the question as to how such limited molecule numbers could exert measurable biological impact. While the molecular function of individual tsRNAs is likely mediated through association with other molecules, addressing the interactome of specific tsRNAs has only been attempted by using synthetic RNA sequences. Since tRNAs carry post-transcriptional modifications, tsRNAs are likely modified but the extent of their modifications remains largely unknown. Here, we developed a biochemical framework for the production and purification of specific tsRNAs using human cells. Preparative scale purification of tsRNAs from biological sources should facilitate experimentally addressing as to how exactly these small RNAs mediate the multitude of reported molecular functions.

## Introduction

Transfer RNAs (tRNAs) are crucial adaptor molecules for the decoding of messenger RNA (mRNA) during protein synthesis. Besides fulfiling this canonical function, tRNAs are also the source of a heterogeneous class of small RNAs, often called tRNA-derived small RNAs (tsRNAs), which have been the subject of intense scrutiny in recent years. An increasing body of work has assigned functional relevance to various tsRNAs because their detection and occurrence are associated with cellular stress and immune responses, cell proliferation and differentiation [1,2], but also ill-understood phenomena such as RNA-based inheritance of extra-chromosomal information between generations [3–6]. tsRNAs have been extensively sequenced resulting in efforts to correctly map and annotate the copy number and sequence identity ofs these small RNAs [7]. However, since tRNAs carry various post-transcriptional modifications, RNA modification-related biases largely disqualify sequencing-based methods from quantifying tRNA and tsRNA abundance [8–12] and, therefore, the full extent of the tsRNA pool in a given biological sample remains largely unknown. Importantly, northern blotting on total RNAs indicated that only 0.1–5% of a given tRNA isoacceptor becomes processed into tsRNAs [13], raising the question as to how such low small RNA quantities could be biologically effective in various and seemingly diverse biological processes.

The biogenesis of different tsRNA species can be attributed to endonucleolytic activities targeting pre-tRNAs or matured tRNAs in the tRNA loop structures (D-, anticodon-, variable-, and T-loops). The best understood mechanism of tsRNA production is stress-induced tRNA fragmentation by anticodon nucleases (ACNases), which is a conserved hallmark of the eukaryotic stress response. Two eukaryotic ACNase protein families (RNase A and T2) specifically cleave matured and full-length tRNAs in response to stress. Mammalian cells express Angiogenin (ANG) [14–16], an RNase A-family enzyme that is kept inactive by binding to its inhibitor, RNH1. Upon stress exposure, RNH1 becomes phosphorylated and releases ANG, which trans-locates from the nucleus to the cytoplasm where the enzyme targets single-stranded RNA sequences in tRNAs with a preference for pyrimidine-purine dinucleotides [17]. ANG cleavage of RNA substrates results in 5ʹ tsRNAs containing a 2ʹ-3ʹ-cyclic phosphate at their 3ʹ-end and 3ʹ tsRNAs containing a 5ʹ-OH moiety. The reproducible production of distinct stress-induced tsRNAs has been reported after starvation [18], oxidative stress [13,19,20], nutritional deficiency [21], hypoxia and hypothermia [22,23], heat shock or irradiation [13,24,25]. While many tRNAs could be ANG substrates, stress-induced ANG activity only affects a fraction of a particular tRNA isoacceptor and isodecoder pool [10,26]. How such limitation is achieved remains unclear. Importantly, tRNAs are the most heavily modified RNAs in any cell type [27,28]. While modifications in the anticodon loop contribute to the optimization of mRNA decoding, modifications that occur outside the anticodon loop (also called core modifications) serve largely structural roles during tRNA processing and maturation [29], but have also been implicated in modulating access of endonucleases. Since stress-induced tsRNAs are likely derived from modified tRNAs, they likely also carry chemical modifications. However, the modification status of individual tsRNAs has not been determined yet. In addition, tsRNA functionality has largely been addressed after re-introducing mostly synthetic RNA sequences into various biological systems, thereby ignoring the potential impact of RNA modifications on tsRNA-mediated silencing of complementary RNA reporters [30], on tsRNA-mediated modulation of embryonic stem cells and early mammalian embryonic development [31,32], on tsRNA-mediated regulation of heterochromatin [33], on tsRNA-mediated suppression of retrotransposons [34,35], on tsRNA-mediated translational enhancement of specific proteins [36] or when probing for protein and RNA binders to specific tsRNA sequences [20,32,37–40]. Predictably, various RNA modifications can affect the hybridization behaviour of RNAs [29,41] or their interactions with proteins [42] and therefore, it would be advisable to utilize modified tsRNAs, rather than synthetic sequences, when addressing and testing their potential for biological impact.

Here, we set out to develop a biochemical framework to purify large amounts of specific tsRNAs, which might allow addressing the biological function of endogenous tsRNAs. As proof of concept, a scalable purification protocol for specific tsRNAs carrying post-transcriptional modifications was established. To this end, two 5ʹ tsRNA species derived from tRNA-Gly^GCC^ and tRNA-Glu^CUC^, which are dominantly featured in tsRNA-related literature, were purified from human cells using chromatographic and hybridization-based methods. The post-transcriptional modification status of purified tsRNAs was determined using LC-MS/MS. To show the applicability of this approach, purified tsRNAs were used for RNA affinity capture experiments and for the approximation of actual copy numbers of specific and stress-induced 5ʹ tsRNAs in a human cell line.

## Results

### Inorganic arsenite-induced tRNA fragmentation coincides with increased cell death

**Figure 1.**
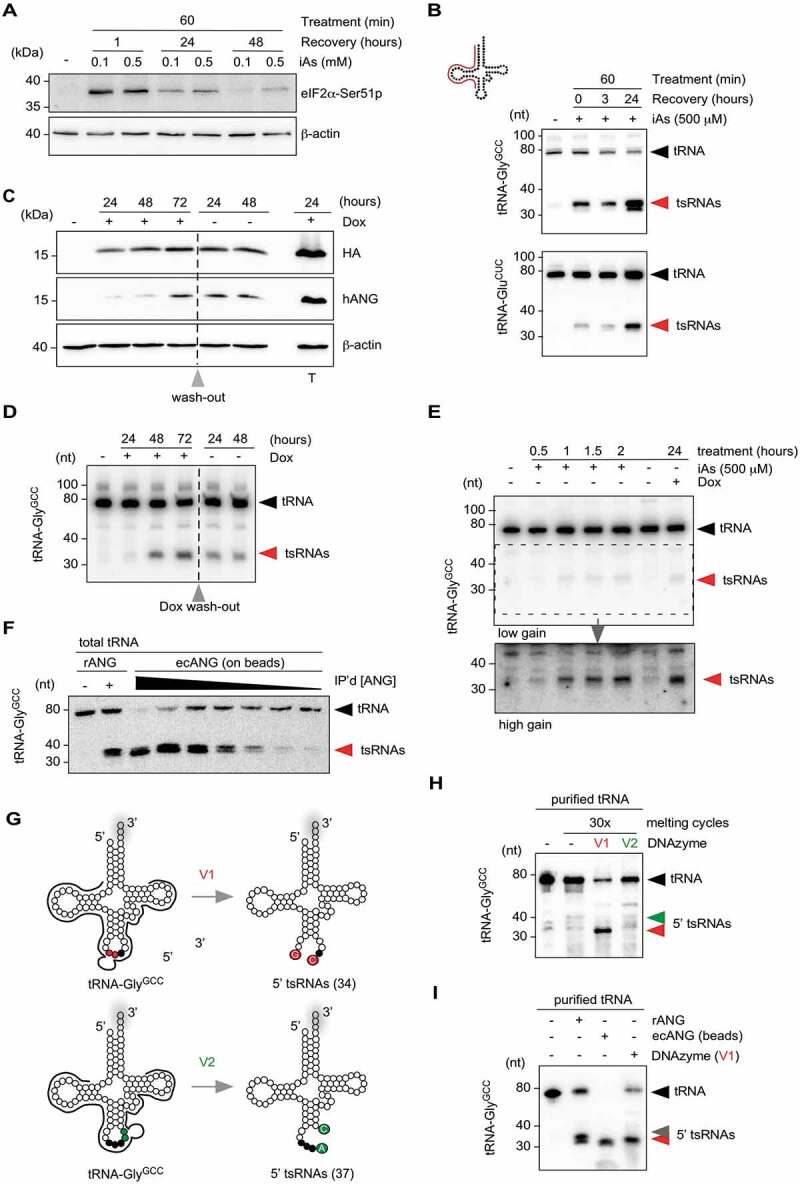
*In vivo* and *in vitro* production of tsRNAs.

5ʹ tsRNA-Gly^GCC^ and 5ʹ tsRNA-Glu^CUC^ are two tsRNA species representing tRNA halves that are often detected in biological samples as very abundant, especially during stress conditions. To produce these tsRNAs, HEK293 cells were treated with inorganic sodium arsenite (iAs), whose impact on cellular stress responses can be monitored by the amount of phosphorylated eukaryotic initiation factor 2α (Fig. 1A). Northern blotting for tRNA-Gly^GCC^ and tRNA-Glu^CUC^ showed tRNA cleavage during the acute stress response to iAs with increased tsRNA levels being detectable 24 h after removing the stressor (Fig. 1B). Of note, dose–response measurements taken 24 h after exposure to acute iAs exposure revealed reduced cell viability exactly at iAs concentrations (between 250 and 500 μM, supplementary Figures 1A and B) that are commonly used as a conduit for the reproducible induction of stress granule formation and tRNA fragmentation. These results indicated limits to using the iAs-mediated oxidative stress response for producing large numbers of tsRNAs without affecting cell viability.

### In vivo tRNA fragmentation using an overexpression system for human ANG

In an attempt to increase tsRNA production, while avoiding excessive stress-induced cell death and overcoming the inhibitory regulation of endogenous ANG, an inducible ANG-expression system was established. Using the Flp-In™-T-REx™ system, human ANG-HA-FLAG (ecANG) was stably inserted into the genome of HEK293 cells. Addition of doxycycline (Dox) induced ecANG expression (Fig. 1C) and resulted in the production of 5ʹ tsRNAs that were comparable in size distribution to iAs-induced 5ʹ tsRNAs (Fig. 1D, E). Importantly, while ecANG localized to visible granules (supplementary Figure 1C) without causing lethality, expression of ecANG affected cell proliferation in a statistically significant manner between 2 and 3 days after constant Dox induction (supplementary Figure 1D). These observations indicated that ectopic expression of ANG could be used for tsRNA production independent of using a stress paradigm. The data also showed that extended ecANG expression, while negatively affecting cell proliferation, was insufficient to fragment the tested tRNA isoacceptor (tRNA-Gly^GCC^) to completion, suggesting effective cellular control mechanisms limiting excessive ANG activity.

### In vitro tRNA fragmentation using secreted ecANG

Recombinant ANG has been used to fragment purified tRNA *in vitro* [22,43]. Since ANG is secreted from cells [44,45], cell culture supernatants from Flp-In™ T-Rex™ 293 cells expressing ecANG were collected and ecANG was immuno-precipitated *via* its FLAG-tag (supplementary Figure 1E). Precipitated ecANG was used for tsRNA production on gel-purified total tRNAs. Northern blotting on the cleavage reactions using probes against the 5ʹ half of tRNA-Gly^GCC^ showed that secreted and immuno-precipitated human Angiogenin can be used to produce scalable quantities of specific tsRNAs *in vitro* (Fig. 1F and supplementary Figure 1F).

### In vitro tRNA fragmentation using DNAzymes

DNAzymes are short deoxyribonucleic acids displaying RNA hydrolysing activity, which can be designed to cleave RNAs with site-directed specificity. As an alternative to ANG-mediated cleavage of tRNAs, DNAzymes of the 10–23 variant [46–48] were designed to target human tRNA-Gly^GCC^ between G34-C35 (variant 1, v1) or A37-C38 (variant 2, v2), respectively (Fig. 1G). Small RNAs were purified from HEK293 cells using ion-exchange chromatography (anIEX) followed by RNA affinity capture using 5ʹ covalently immobilized amino-modified DNA oligonucleotides complementary to the 5ʹ end of tRNA-Gly^GCC^. Purified tRNA-Gly^GCC^ was subjected to DNAzyme activity *in vitro*. Northern blotting using probes against the 5ʹ half of tRNA-Gly^GCC^ showed almost quantitative hydrolysis into tsRNAs when using v1 while v2 did not hydrolyse tRNA-Gly^GCC^ to an appreciable extent (Fig. 1H). Of note, previous attempts targeting the phosphodiester bond between positions 37–38 in a different tRNA (*Drosophila melanogaster* tRNA-Asp^GUC^) yielded also limited cleavage [48] indicating a common interference with DNAzyme efficiency at exactly this position. While the purine-pyrimidine context at this position in both human tRNA-Gly^GCC^ and *Drosophila* tRNA-Asp^GUC^ is ApC, this dinucleotide is the least addressable context for DNAzymes according to (46). In addition, C38 in both tRNAs is modified by Dnmt2/TRDMT1 proteins, which might negatively affect cleavage yield as has been observed for other RNA modifications [49]. These observations indicated that while DNAzymes could be used as tools for tRNA fragmentation, the existence of particular RNA modifications in tRNAs purified from endogenous sources might impact DNAzyme reaction efficiency, which necessitates the informed design of DNAzymes that target tRNAs at unmodified positions.

### Comparison of approaches used for in vitro tRNA fragmentation

When comparing the tRNA fragmentation efficiencies mediated by recombinant ANG, immuno-purified ecANG or by DNAzyme v1, ecANG was able to quantitatively fragment tRNA-Gly^GCC^ (Fig. 1I). Of note, while DNAzyme v1 produced 5ʹ tsRNAs migrating with expected mobility (34 nts), *in vitro*-targeting of recombinant ANG or immunoprecipitated ecANG to tRNA-Gly^GCC^ resulted in 5ʹ tsRNAs migrating with slightly different mobility (Fig. 1I). ANG hydrolyses preferentially CpA in single-stranded RNA, but also CpC and CpG dinucleotides can be targeted [17,50,51]. Human tRNA-Gly^GCC^ contains C35-C36, C36-A37 and a C38-G39 in the anticodon and ANG-mediated creation of two closely migrating 5ʹ tsRNAs could be the result of structural changes caused by *in vitro* melting and refolding of tRNAs or by differences in the activities of recANG and ecANG. In addition, ANG cleavage creates 5ʹ tsRNAs containing a 2ʹ-3ʹ-cyclic phosphate (cP) at their 3ʹ ends, which increases their migration behaviour. Importantly, two distinct 5ʹ tsRNA species could sometimes be observed also *in vivo*, especially during the stress recovery or when exposing cells to exceedingly high iAs concentrations (Fig. 1, supplementary Figure 1B and Figures below), which could have resulted in stochastic cP-ring opening since cP is susceptible to background hydrolysis in aqueous solutions [52]. These observations indicated that the outcomes of applying *in vitro* methodology for the production of specific tsRNAs can differ in respect to efficiency and identity of the resulting tsRNAs.

### Purification of specific stress-induced and ectopic ANG-produced tsRNAs

**Figure 2.**
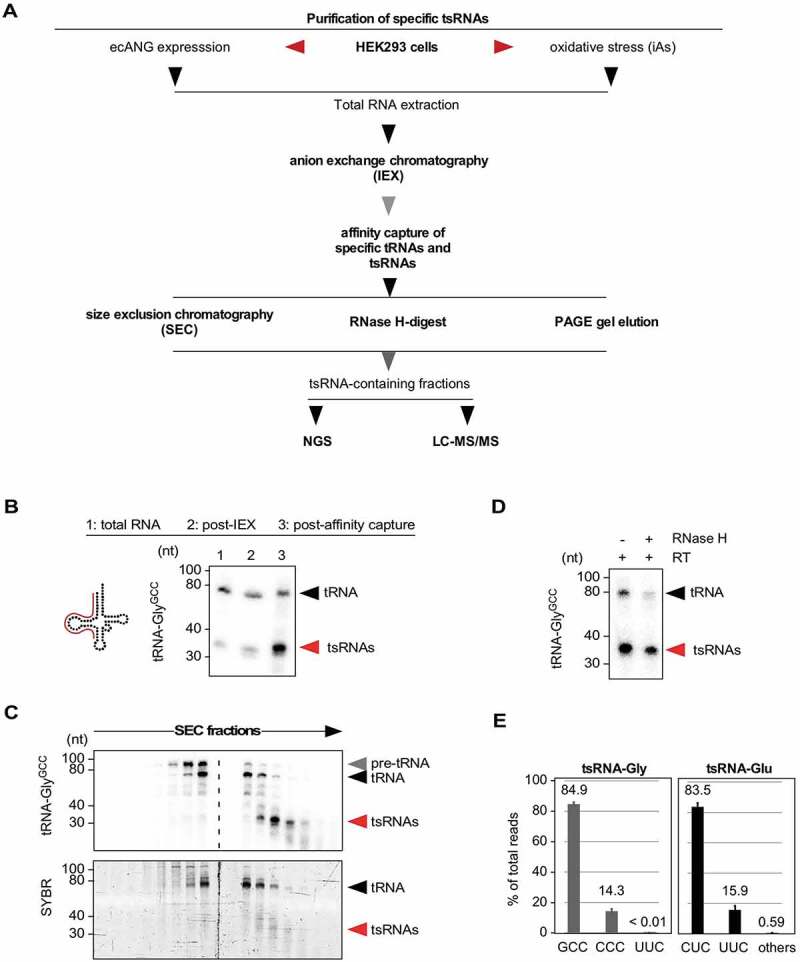
Purification of specific tsRNAs.

To produce tsRNAs in scalable quantities from endogenous sources, 5ʹ tsRNA-Gly^GCC^ and 5ʹ tsRNA-Glu^CUC^ were purified from iAs-treated or ecANG-expressing Flp-In™ T-Rex™ 293 cells by anIEX and affinity capture using 5ʹ amino-modified DNA oligonucleotides (complementary to the target RNA) covalently immobilized on NHS-linked sepharose columns (Fig. 2A). Denaturing PAGE and northern blotting for target tRNAs (i.e., tRNA-Gly^GCC^) showed clear enrichment of 5ʹ tsRNAs after the affinity capture step (Fig. 2B). To separate 5ʹ tsRNAs from residual co-purified parental tRNAs, preparative SEC (prepSEC), RNaseH-mediated RNA removal or urea-PAGE gel elution was used (Fig. 2C, D). Both prepSEC and gel elution followed by RNA precipitation resulted in very reproducible tsRNA content. The calculated yield of such an RNA purification indicated that about 1–2 μg of a particular 5ʹ tsRNA species (about 90–180 pmoles) can be purified from the small RNA fraction (ca. 200 μg RNA) obtained from about 100 million HEK293 cells (ca. 4 mg of total RNA) after exposure to iAs or expression of ecANG.

### Determining the identity of purified 5ʹ tsRNAs

Northern blotting of purified 5ʹ tsRNA-Gly^GCC^ for other tRNA sequences showed very low cross-reactivity with 3ʹ tsRNA-Gly^GCC^ or 5ʹ tsRNA-Glu^CUC^ sequences indicating low contamination of the affinity capture eluate with other tRNA sequences (supplementary Figure 2A). Triplicate small RNA sequencing of ecANG-produced 5ʹ tsRNA-Gly^GCC^ revealed that 84.9% of all reads mapped to 5ʹ tsRNA-Gly^GCC^ while 14.3% were derived from the 5ʹ end of the isoacceptor tRNA-Gly^CCC^ (particularly from tRNA-Gly^CCC−1.1^ and tRNA-Gly^CCC−1.2^, which share 100% sequence identity with the 5ʹ half of tRNA-Gly^GCC^, Fig. 2E and supplementary Figure 2B). Similarly, after purification of 5ʹ tsRNA-Glu^CUC^ from ecANG cells, 83.5% of all reads mapped to 5ʹ tsRNA-Glu^CUC^ while 15.9% were derived from the 5ʹ end of the isoacceptor tRNA-Glu^UUC^, which shares 96% sequence identity with the 5ʹ half of tRNA-Glu^CUC^ (Fig. 2E and supplementary Figure 2B). These results indicated that purification of particular 5ʹ tsRNAs can be achieved with only minor contamination from other RNAs including other tRNA-derived sequences although we cannot absolutely exclude the co-purification of additional RNAs since sequencing-based RNA identification approaches suffer from amplification biases caused by particular RNA modifications (such as m^1^A), which often interfere with reverse transcription [12], thereby leading to an underrepresentation of particular sequencing reads and therefore an underestimation of RNA existence in the biological sample. Since the hybridization-based purification of particular tRNA-Gly and tRNA-Glu isoacceptors produced about 15% co-purified tRNA isoacceptor sequences (Gly^CCC^ and Glu^UUC^, respectively), we will, from here on, label tRNA-derived sequences as tRNA or 5ʹ tsRNA for Gly^GCC/CCC^ and Glu^CUC/UUC^.

### Determining the modification status of purified tsRNAs

**Figure 3.**
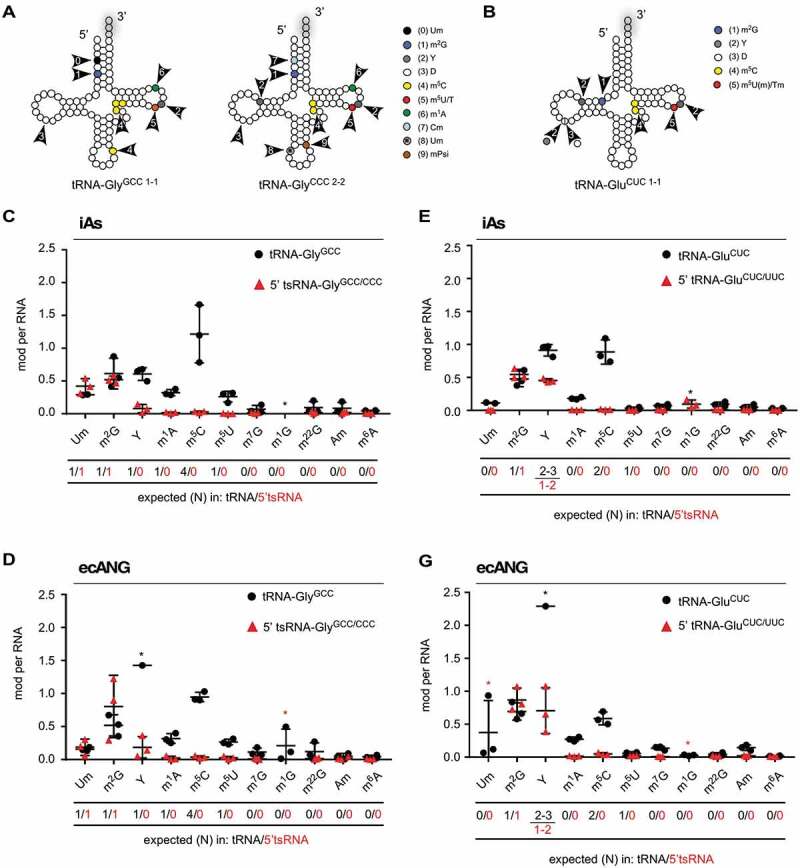
Determination of RNA modification patterns in purified tsRNAs.

LC-MS/MS analyses can reproducibly quantify individual RNA modifications in purified tRNAs as shown by analysing six LC-MS/MS measurements on commercially available tRNA-Phe from *Saccharomyces cerevisiae* (supplementary Figure 3A and B confirming the analysis of *S.c*. tRNA-Phe published in [53]). 5ʹ tsRNA-Gly^GCC/CCC^ and 5ʹ tsRNA-Glu^CUC/UUC^ along with their remaining parental full-length tRNAs were purified from biological triplicate experiments after either iAs exposure or ecANG expression followed by LC-MS/MS analyses (Fig. 3, supplementary Tables 1 and 2). Absolute quantification of a subset of modifications in full-length tRNAs and 5ʹ tsRNAs revealed modifications predicted to reside in the 5ʹ halves of parental tRNAs (N2ʹ-methylguanosine, m^2^ G in both 5ʹ tsRNAs and 2ʹ-O-methyluridine, Um in 5ʹ tsRNA-Gly^GCC/CCC^) [27] albeit consistently with low stoichiometry indicating that both modified and unmodified tRNAs and their 5ʹ tsRNA-derivatives were purified. Importantly, RNA modifications not reported in these tRNAs were either not present or detected at very low levels (i.e., m^7^ G, m^1^ G, m^22^ G, Am, m^6^A) in targeted RNA species (Fig. 3C–G, supplementary Table 2). Furthermore, levels of tRNA modifications predicted to reside in the 3ʹ halves of full-length tRNAs (3ʹ of the ANG cleavage sites in the anticodon loop) such as m^5^ C, m^1^A or m^5^ U(m) were very low to non-existent in purified tsRNAs (Fig. 3C–G, supplementary Table 2). Of note, quantification of dihydrouridine (D) was not possible due to the presence of D in the digestion cocktail introduced through the deaminase inhibitor tetrahydrouridine. These combined results indicated that tsRNA species purified from cells after iAs-exposure or expression of ecANG consisted of a mix of RNA molecules with and without particular RNA modifications.

**Figure 4.**
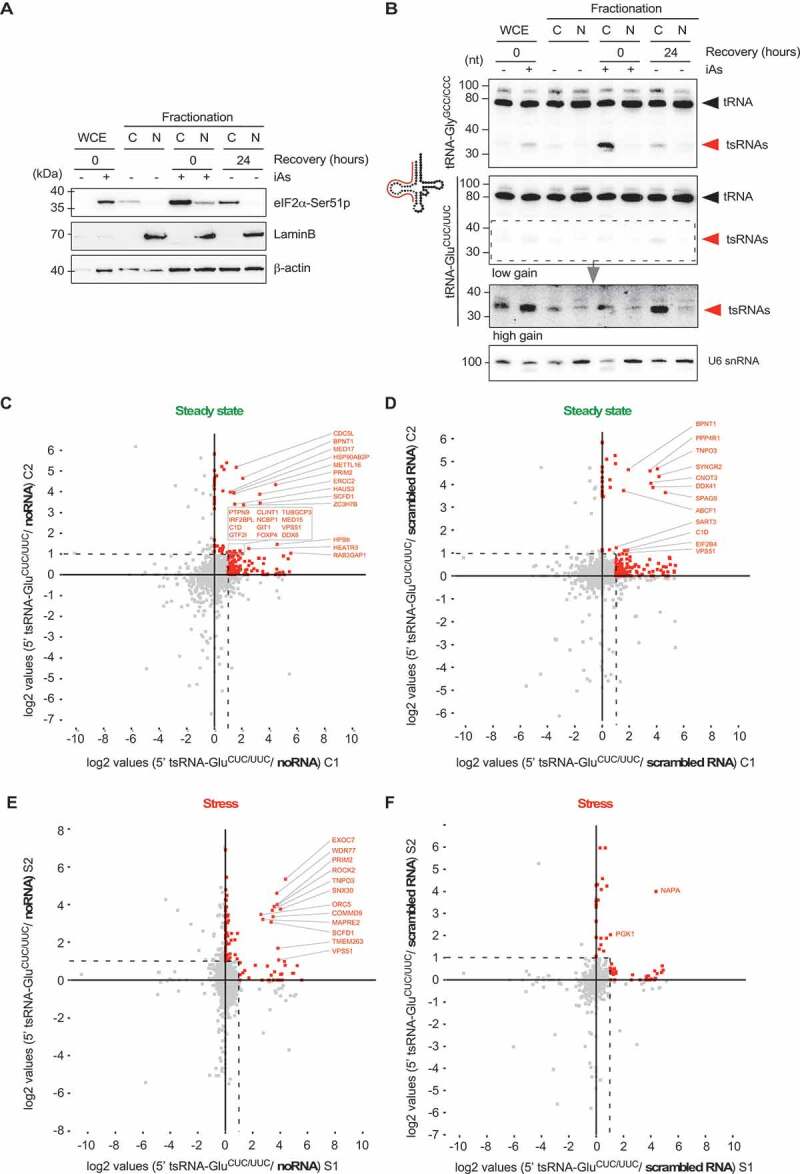
RNA affinity capture of proteins using purified tsRNAs.

**Figure 5.**
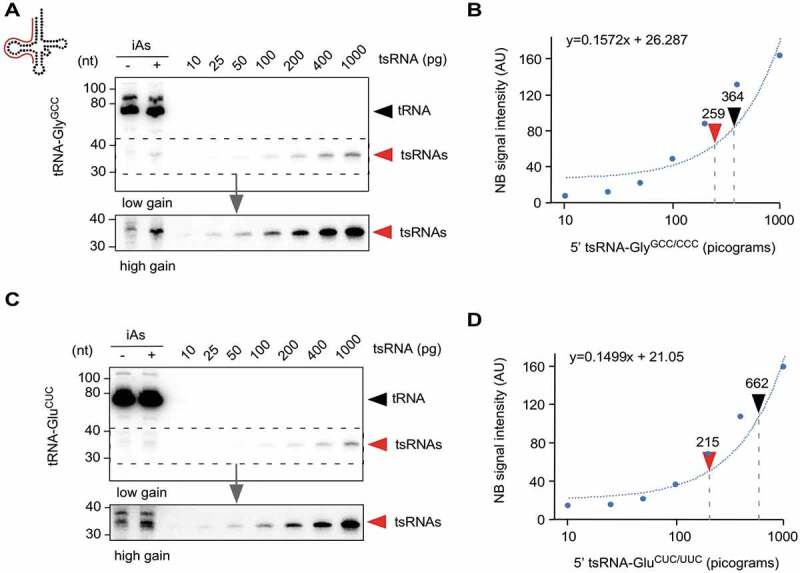
Semi-quantification of tsRNA numbers using purified tsRNAs.

### Identification of proteins associating with endogenous 5ʹ tsRNAs

RNA affinity capture of interacting proteins from cell extracts was performed using 5ʹ tsRNA-Glu^CUCUUC^ purified from Flp-In™ T-Rex™ 293-ecANG cells. To this end, 5ʹ tsRNA-Glu^CUC/UUC^ was 5ʹ biotinylated and coupled to streptavidin-coated sepharose beads. Since virtually all iAs-induced 5ʹ tsRNA-Gly^GCC/CCC^ and 5ʹ tsRNA-Glu^CUC/UUC^ resided in the cytoplasm (Fig. 4A, B), fractionated cytoplasmic protein extracts (CPEs) from HEK293 cells growing under steady-state conditions or from cells subjected to acute iAs exposure were used for RNA affinity capture experiments. Mass spectrometry analysis of tryptic digests and label-free quantification (LFQ) of peptides was performed against empty streptavidin matrix (noRNA control) and an unrelated synthetic and unmodified RNA control (scrambled). The latter was introduced to differentiate between proteins that were associating specifically with 5ʹ tsRNA-Glu^CUC/UUC^ and those that were general RNA binders without specificity to 5ʹ tsRNA-Glu^CUC/UUC^. Label-free MS yielded a total of 1107 quantified proteins with at least one razor and unique peptide and a minimum of three LFQ values out of 12 experiments (supplementary Table 3). Two replicate experiments probing CPEs collected during steady-state conditions yielded 171 proteins with a positive fold change (log_2_ ratio ≧ 0) in both, and at least a twofold change (log_2_ ratio ≧ 1) in one of the replicates when normalized to noRNA control, and 168 proteins when normalized to the scrambled RNA control experiments (Fig. 4C, D, supplementary Figures 4A and B, supplementary Table 3). Furthermore, LFQ analysis of peptides obtained from probing CPEs collected after iAs-exposure yielded 104 proteins when normalized to noRNA control, and 56 proteins when normalized to the scrambled RNA control experiments again with a fold change ≧1 in both (log_2_ ratio ≧ 0), and at least a twofold change (log_2_ ratio ≧ 1) in one of the replicates (Fig. 4E, F, supplementary Figure 4A and B, supplementary Table 3). These data revealed specific protein associations with 5ʹ tsRNA-Glu^CUC/UUC^ but not with a generic RNA sequence and suggest that stress conditions might lead to changes in tsRNA-associated proteins.

### Semi-quantification of tsRNA copy numbers per cell using purified tsRNAs

RNA modification-related biases in efficient reverse-transcription of tRNA-derived sequences, including various mapping issues, largely disqualifying sequencing-based methods from quantifying tRNA and tsRNA abundance [8–11]. Hence, the actual copy number of individual tRNAs or specific tsRNAs in any cell type under specific growth or stress conditions remains largely unknown. Microscale thermophoresis has been recently used to measure copy numbers of endogenous tRNAs [54]. As for tsRNAs, copy number estimates for particular tsRNA species have been attempted based on textbook calculations (for instance, in supplementary data in [20]), but these estimates remain experimentally unproven. In an attempt to approximate the copy number of specific tsRNAs in iAs-exposed HEK293 cells, 5ʹ tsRNA-Gly^GCC/CCC^ and 5ʹ tsRNA-Glu^CUC/UUC^ levels were determined semi-quantitatively using northern blotting. To this end, total RNA extracted from a defined number of stressed cells was probed along with a dilution series of purified endogenously modified tsRNAs. Radiographic signals from serial tsRNA dilutions were plotted as standard curve and quantified to calculate the relative mass of individual stress-induced tsRNAs species per cell. When measuring the average yield of total RNA after Trizol extraction, the calculation indicated that this extraction method yielded about 4.75 picograms RNA from one million HEK293 cells (according to NanoDrop quantification). Blotting 4.5 μg of total RNA from HEK293 cells before and after iAs exposure against a dilution of purified 5ʹ tsRNAs resulted in signals that could be semi-quantitatively and comparatively quantified. Ensuing calculations indicated that the copy number of tsRNAs in a single HEK293 cell after iAs-induced stress was about 14.000 for 5ʹ tsRNA-Gly^GCC/CCC^ (Fig. 5A, B) and about 12.000 for 5ʹ tsRNA-Glu^CUC/UUC^ Fig. 5C, D).

## Discussion

tRNA-derived small RNAs (tsRNAs) can be extracted from many small RNA sequencing data sets. The biological significance of these small RNAs has been ignored until recently because their varying abundance and heterogeneity made them likely remnants of tRNA maturation or tRNA degradation intermediates. The potential for biological impact of specific tsRNAs has only been recognized after discovering that stress-induced tRNA fragmentation resulting in tRNA halves is a conserved part of the eukaryotic stress response with particular effects on protein translation and cellular survival [13,19,20,55]. Ever since, an increasing number of reports has been assigning particular functions to specific tsRNAs suggesting rather defined molecular impact on all kinds of cellular processes, which are not necessarily limited to the cellular stress response. However, actual data on the mechanistic details as to how tsRNAs impact specific cellular processes remain scarce. For instance, it is unclear whether the few per cent of isoacceptor-specific tsRNAs act as single entities or rather in bulk with other tsRNAs. Furthermore, many of the current reviews re-iterate mere correlations by connecting tsRNA detection and abundance (mostly obtained from RNA sequencing data that have not been controlled for RNA modification-specific biases) with a wide range of cellular pathways, often without mentioning that the mechanistic underpinnings of tsRNA function have not yet been addressed (critically discussed in [1]).

To better understand the mechanistic details of tsRNA function, improved methodology needs to be developed, which would allow measuring actual tsRNA copy numbers, localizing specific tsRNAs *in situ*, mapping tsRNA modification patterns and calculating their stoichiometry, as well as determining the molecular interactions of specific tsRNAs. The experimental basis for addressing most of these questions should be the availability of pure tsRNA sequences, preferably from cellular sources and conditions where and under which tsRNAs are being produced. This is important because tRNAs are the most highly modified tRNAs in any cell type, making it likely, but largely untested, that the functionality of specific tsRNAs depends on their modification state. Biochemical attempts for enriching small RNAs including tsRNA have been published [56]. However, reports that aimed at addressing molecular function or interactions of specific tsRNAs have almost exclusively used synthetic RNA sequences (with published exceptions [57]) thereby largely ignoring the possibility that tsRNA structure and function could be determined by RNA modifications. Theoretically, chemical RNA synthesis allows introducing modified nucleotides at specific positions given that these positions are known once such modification patterns have been determined. However, practically, many modified nucleotides remain commercially unavailable, which necessitates (often) complicated chemical synthesis by expert laboratories. Furthermore, commercially available (unmodified) RNAs appear to contain trace amounts of modified nucleotides [58], which might affect experimental outcomes when testing synthetic or synthetically modified tsRNAs for structure–function relationships.

A potentially viable alternative is the purification of tsRNAs from endogenous sources, especially under *in vivo* conditions that promote tRNA fragmentation or after *in vitro* processing of purified tRNAs. This alternative is rooted in the previous success to purify individual tRNAs and systematically determine their modification patterns after hybridization-based affinity capture of target RNAs from complex samples followed by LC-MS/MS [59].

Here, we report on different *in vitro* and *in vivo* strategies for the production of tsRNAs carrying RNA modifications. While some of the *in vitro* approaches would allow quantitative fragmentation of input RNAs into tsRNAs, use of resulting tsRNAs obtained from these approaches needs to be matched with planned downstream applications. This might become important for planning specific experiments since denaturing and re-folding of tRNAs (serving as substrates for DNAzymes or purified ANG) might result in tsRNA identities that are different from stress-induced tsRNAs produced in a cellular environment and thereby affect experimental outcomes. Furthermore, our findings that particular tRNA positions carrying RNA modifications are not addressable by DNAzymes are informative since use of such DNAzymes would result in tsRNAs with particular modification patterns that are different from the once produced in a cellular context through the activity of particular endonucleases.

On the other hand, the presented *in vivo* approaches, which employ either endogenous or ectopic ANG activity in living cells, resulted in tsRNAs that were homogeneous in sequence identity but rather heterogeneous in RNA modification content. Quantitative RNA mass spectrometry for a select number of modifications in the HEK293 cells revealed that both purified tsRNAs and co-purified tRNAs were not quantitatively modified (expected mods/detected mods). These results could be explained by biological context but could also be rooted in technical bias introduced during the presented workflow for the purification of tRNA-derived sequences. For example, while database entries list particular mature tRNAs as modified at particular positions [27], the extent and stoichiometry of particular modifications in a given tRNA-isoacceptor or tRNA-isodecoder pool purified from different cell types has not been systematically determined. Of note, fast dividing cells such as cancer-derived cell lines showed upregulation of tRNA expression and deregulation of various tRNA modification enzymes [60,61], yet some tRNAs in cancer cells exhibited hypo-modification at specific positions [62] indicating that rapidly dividing cells might not always quantitatively introduce all RNA modifications or that absence of particular RNA modifications might be advantageous depending on the cell type and growth conditions. The latter point is supported by the existence of tRNA-demethylating activities [63,64] indicating that specific positions in individual tRNAs can be variably modified. Importantly, partial tRNA modification has been suggested to play a role in stress-induced reprogramming of protein translation [65,66]. Alternatively, overexpression of ANG outside stress response circuitry might cause ectopic tRNA fragmentation thereby resulting in tsRNAs originating from incompletely modified tRNAs.

In addition, also technical bias could have resulted in the affinity purification of a mix of modified, hypo-modified and non-modified tRNA-derived sequences. Since we employed hybridization-based affinity capture methodology with the aim of purifying specific tsRNAs from human cells exposed to stress conditions or after ectopically expressing the mammalian anticodon nuclease ANG, the use of DNA oligonucleotides matching an unmodified and not a modified tRNA-derived sequence might have affected the output of the purification procedure because of different hybridization efficiencies, which can be determined by RNA modifications.

In summary, the presented methods provide a modular framework for the systematic purification of specific tsRNAs from endogenous sources which could be used when addressing various unresolved aspects in tsRNA biology. As laid out in some of our follow-up experiments, purified tsRNAs containing quantifiable modification patterns in particular stoichiometry can be used for *in vitro* protein capture experiments, which, in combination with experiments using synthetic tsRNA sequences, could help addressing the impact of modifications on protein binding. Furthermore, purified tsRNAs could be used for the development of amplification-independent tsRNA quantification methods, which, in combination with using synthetic tsRNA sequences, might allow determining the effects of RNA modifications on hybridization-based read-outs. The values that we have calculated after northern blotting as to how many individual stress-induced tsRNAs might exist in a single cell fit well to previously obtained values (supplemental information in [20]) although such quantifications are inherently dependent on the initial RNA concentration measurements, the outcome of which can differ by magnitudes depending on the measurement method used [67,68]. In addition, the recently reported possibility of intra- and intermolecular cross-hybridization between 5ʹ tsRNA-Gly^GCC^ and 5ʹ tsRNA-Glu^CUC^ in extracellular space [69] could be tested for modification-dependence using purified tsRNA species from extra-cellular fluids. Furthermore, reported tsRNA activities as *bona fide* small RNA entities with post-transcriptional gene regulatory functions akin to siRNAs or miRNAs [30,35] will need to be tested using endogenously produced tsRNAs since various RNA modifications affect the base-pairing capabilities and therefore might modulate the activities that were originally reported for synthetic tsRNAs. Lastly, the recently reported effect of RNA modifications on the efficiency of sperm-borne small RNAs (including tsRNAs) for intergenerational transmission of paternally acquired metabolic disorders [70,71] raises the question as to which RNAs exactly contribute to these processes. The present consensus points towards a role for sperm-carried tsRNAs but the identity, exact modification status of individual tsRNA species and their abundance has not been addressed in molecular detail.

Taken together, while the described approaches lay the groundwork for the reproducible purification of specific tsRNA species in scalable fashion, these strategies represent basic workflows, which will need to be optimized, adjusted and extended depending on the application and type of experiment that will utilize purified tsRNAs.

## Supplementary Material

Supplemental Material

## Accession numbers

Small RNA sequencing has been deposited at NCBI GEO database under accession number GSE139124.
