# Supplementary material for: Production and purification of endogenously modified tRNA-derived small RNAs
Source: RNA Biol. 2020 Mar 5;17(8):1104–15. doi: 10.1080/15476286.2020.1733798 (PMC7549616; doi:10.1080/15476286.2020.1733798)
Supplement: Supplemental Material [file KRNB_A_1733798_SM0715.zip › Supplementary information/STable 1R.pdf]

Drino_ Supplementary Table 1

Compound	Precursor Ion	MS1 Res	Product Ion	MS2 Res	Ret Time [min]	Fragmentor [V]	Collision Energy [eV]
A	268.1	Wide	136	Unit	5.1	200	25
A SILIS	283	Wide	146	Unit	5.1	200	25
Am	282.1	Wide	136	Unit	6.1	130	17
Am SILIS	298	Wide	146	Unit	6.1	130	17
C	244.1	Wide	112	Unit	2	200	20
C SILIS	256	Wide	119	Unit	2	200	20
G	284.1	Wide	152	Unit	4.1	200	25
G SILIS	299	Wide	162	Unit	4.1	200	25
m1A	282.1	Wide	150	Unit	2.3	150	25
m1A SILIS	298	Wide	161	Unit	2.3	150	25
m1G	298.1	Wide	166	Unit	4.8	105	13
m1G SILIS	314	Wide	177	Unit	4.8	105	13
m22G	312.1	Wide	180	Unit	5.6	105	13
m22G SILIS	329	Wide	192	Unit	5.6	105	13
m2G	298.1	Wide	166	Unit	5	95	17
m2G SILIS	314	Wide	177	Unit	5	95	17
m5C	258.1	Wide	126	Unit	3.5	185	13
m5C SILIS	271	Wide	134	Unit	3.5	185	13
m5U	259.1	Wide	127	Unit	4.2	95	9
m5U SILIS	271	Wide	134	Unit	4.2	95	9
m6A	282.1	Wide	150	Unit	6.5	125	17
m6A SILIS	298	Wide	161	Unit	6.5	125	17
m7G	298.1	Wide	166	Unit	3.3	100	13
m7G SILIS	314	Wide	177	Unit	3.3	100	13
U	245.1	Wide	113	Unit	2.9	95	5
U SILIS	256	Wide	119	Unit	2.9	95	5
Um	259.2	Wide	113	Unit	4.4	96	8
Um SILIS	271.1	Wide	119	Unit	4.4	96	8
Y	245.1	Wide	209	Unit	1.7	90	5
Y SILIS	256	Wide	220	Unit	1.7	90	5
