# Supplementary material for: Production and purification of endogenously modified tRNA-derived small RNAs
Source: RNA Biol. 2020 Mar 5;17(8):1104–15. doi: 10.1080/15476286.2020.1733798 (PMC7549616; doi:10.1080/15476286.2020.1733798)
Supplement: Supplemental Material [file KRNB_A_1733798_SM0715.zip › Supplementary information/STable 2R.pdf]

iAs

	tRNA Gly-1	tRNA Gly-2	tRNA Gly-3	tsRNA Gly-1	tsRNA Gly-2	tsRNA Gly-3	tRNA Glu-1	tRNA Glu-2	tRNA Glu-3	tsRNA Glu-1	tsRNA Glu-2	tsRNA Glu-3
Um	0,29560801		0,28684211	0,41349737	0,54161162	0,31247111		0,1068281	0,11878771	0,00048628	0,00332907	0,00140809
m2G	0,54814731	0,87347984	0,41842105	0,58042779	0,51005431	0,46731996	0,60874258	0,44707558	0,37751712	0,63435821	0,50564934	0,49651424
Psi	0,4959428	0,67719223	0,64605263	0,020292	0,14604433	0,07303319	0,81079331	0,96946497	0,95572581	0,48433943	0,43080682	0,44203198
m1A	0,37587244	0,28952422	0,30328947	0,00484966	0,02421841	0,01941389	0,20205073	0,17786878	0,16814699	0,00972569	0,00480866	0,00639056
m5C	1,66467117	0,78515042	1,19605263	0,01250702	0,03522677	0,03512989	0,73826228	1,09338556	0,82554749	0,01774939	0,00838433	0,01158966
m5U	0,3151847	0,16684446	0,30131579	0,00063811	0,00660502	0,00138671	0,01813276	0,03258257	0,04881687	0,00291771	0,0001233	0,00303281
m7G	0,02871248	0,13740132	0,04078947	0,00370106	0,02054895	0,0143293	0,09325418	0,05394819	0,06780121	0,00753741	0,00369897	0,00498247
m1G				0,001					0,0012	0,0442	0,0787	0,1645
m22G	0,0358906	0,20119479	0,04802632	0,00523253	0,03008953	0,02033836	0,1295197	0,06623342	0,08678554	0,01069826	0,00493196	0,00530742
Am	0,02479714	0,18647322	0,04407895	0,00459442	0,02788786	0,02449847	0,09066379	0,0197632	0,04339277	0,01045512	0,00456206	0,00758202
m6A	0,04828917	0,03925752	0,05065789	0,00063811	0,0080728	0,00231118	0,03367512	0,02830945	0,03145976	0,00121571	0,0006165	0,00097483

ecANG

	tRNA Gly-1	tRNA Gly-2	tRNA Gly-3	tsRNA Gly-1	tsRNA Gly-2	tsRNA Gly-3	tRNA Glu-1	tRNA Glu-2	tRNA Glu-3	tsRNA Glu-1	tsRNA Glu-2	tsRNA Glu-3
Um	0,14787485	0,13453238	0,18572594	0,19811395	0,05574847	0,30295211	0,12037109	0,0665521	0,93440318			
m2G	0,35283123	0,52931485	0,67291641	0,89636868	0,29171024	1,2234365	0,83607194	0,66226488	0,58040767	0,80740652	0,72363281	1,07104837
Psi			1,42515643	0,13962744	0,36419559	0,05162786			2,29120426	0,65226147	0,38631076	1,07697828
m1A	0,25254462	0,30410553	0,39862956	0,00784191	0,05598071	0,00613558	0,3055795	0,27160965	0,24115019	0,01818351	0,00971101	0,02118258
m5C	0,90021374	1,03012182	0,91225084	0,03156662	0,05635556	0,02011205	0,50360295	0,56540333	0,69290973	0,03723759	0,03940481	0,07041207
m5U	0,23130119	0,31284757	0,25265908	0,02354001	0,04791899	0,00439129	0,0231189	0,071492	0,0683735	0,02343906	0,01984596	0,01915426
m7G	0,07259732	0,0837392	0,18202531	0,00243188	0,01842332	0,00208065	0,15756443	0,15048643	0,10335008	0,00650504	0,00430749	0,01107916
m1G	0,11524738	0,01434196	0,4957748				0,03141267	0,03686564	0,01729083			
m22G	0,07278896	0,01762931	0,27039675	0,01020171	0,02600582	0,00226228	0,03342276	0,02936321	0,07029088	0,0086519	0,01468888	0,02442693
Am	0,05540978	0,09035019	0,00663629	0,01275039	0,04296532	0,00374186	0,18395092	0,14681603	0,10056844	0,00839067	0,02781475	0,0259476
m6A	0,0351445	0,02315662	0,06682964	0,00638078	0,01208399	0,00142595	0,01432404	0,0146816	0,02120002	0,00499797	0,00873754	0,01236703
