# Supplementary material for: Production and purification of endogenously modified tRNA-derived small RNAs
Source: RNA Biol. 2020 Mar 5;17(8):1104–15. doi: 10.1080/15476286.2020.1733798 (PMC7549616; doi:10.1080/15476286.2020.1733798)
Supplement: Supplemental Material [file KRNB_A_1733798_SM0715.zip › Supplementary information/Supplementary Material_MS.docx]

**Supplementary Figure legends**

**Supplementary Figure 1. Comparison of cell viability during tsRNA production.**

(A) Lethal dose (LD_50_) determination of HEK293 cells after exposing to different iAs concentrations. Triplicate cell viability measurements 24 h after iAs exposure and wash-out indicate an LD_50_ value of 333 µM (red arrowhead), a concentration of iAs that is still below the commonly used iAs concentration (500 µM, black arrowhead), which would correspond to a LD_61_ value.

(B) Northern blotting of total RNA (3 µg) from HEK293 cells exposed to different iAs concentrations (LD_20_ to LD_60_) using 5’ probes against tRNA-Gly^GCC^ and tRNA-Glu^CUC^ (annealing of probe in full-length tRNA according to cartoon). Black arrowhead: full-length tRNAs; red arrowhead: tsRNAs.

(C) Indirect immuno-fluorescence images of Flp-In™ T-Rex™ 293-ecANG cells before and after ecANG expression using Dox using antibodies against human ANG. Red: DNA; green: ANG.

(D) Triplicate cell viability measurements of Flp-In™ T-Rex™ 293-ecANG cells over the course of Dox-mediated ecANG expression. Statistical analysis was performed using a Student’s t-test (equal variance).

(E) Western blotting on a dilution series of recombinant ANG and immuno-precipitated ANG from Flp-In™ T-Rex™ 293-ecANG-derived cell culture medium (1 mL collected 48 h after Dox-induction) using antibodies against human ANG (hANG). Immuno-precipitated ecANG on beads was washed with buffers of different stringency (NaCl concentration) and different volumes of the immuno-precipitated eluate were analysed to determine the mass of ecANG that can be precipitated from a given volume of cell culture medium.

(F) Northern blotting on recombinant ANG-mediated or ecANG-mediated *in vitro* tRNA fragmentation after purification of total tRNAs under native conditions using acidic phenol and native gel extraction followed by no melting and re-annealing of tRNA fraction (condition A) or melting and reannealing of tRNA fraction (condition B) or purification of total tRNA using Trizol followed by denaturing gel elution, melting and reannealing of tRNA fraction (condition C). The blot was hybridized with a 5’ probe against tRNA-Gly^GCC^. Black arrowhead: full-length tRNAs; red arrowhead: tsRNAs.

**Supplementary Figure 2**

(A) SYBR staining of urea-PAGE after small RNA enrichment using LiCl precipitation (LiCl enrichment) and affinity capture of tRNA-Gly^GCC^ -derived sequences from the LMW RNA pool (in triplicate). Northern blotting of total RNA, LiCl-enriched LMW RNA and affinity captured tRNA- and 5’ tsRNA-Gly^GCC^ using probes against the 5’ portion of tRNA-Gly^GCC^, the 3’ portion of tRNA-Gly^GCC^ and the 5’ portion of tRNA-Glu^CUC^. Black arrowheads: full-length tRNAs; red arrowheads: tsRNAs.

(B) Sequences of full-length tRNA-Gly^GCC^, tRNA-Gly^CCC-1.1^, tRNA-Glu^CUC^ and tRNA-Glu^UUC-3.1^(according to <http://gtrnadb.ucsc.edu/>), which could be captured by the used NHS-coupled complementary oligonucleotides (depicted in red).

**Supplementary Figure 3**

(A) LC-MS/MS chromatogram of an exemplary calibration measurement preceding the analysis of tRNA-derived sequences.

(B) Results from sextuplicate LC-MS/MS analysis of tRNA-Phe purified from yeast (*Saccharomyces cerevisiae*. S.c.). From six replicate measurements, plotted as single data points with mean and standard deviation.

Abbreviations: Y (pseudouridine), C (cytidine), m^1^A (1-methyladenosine), U (uridine), m^7^G (7-methylguanosine), m^5^C (5-methylcytidine), m^3^C (3-methylcytidine), Cm (2’-O-methylcytidine), G (guanosine), Gm (2’-O-methylguanosine), m^5^U (5-methyluridine), Um (2’-O-methyluridine), m^1^G (1-methylguanosine), m^2^G (2-methylguanosine), A (adenosine), I (inosine), m^22^G (2,2-dimethylguanosine), Am (2’-O-methyladenosine), m^6^A (6-methyladenosine), t^6^A (N6-threonylcarbamoyladenosine).

**Supplementary Figure 4**

(A) Heatmaps depicting protein associations with 5’ tsRNA-Glu^CUC/UUC^ (purified from Flp-In™ T-Rex™ 293-ecANG cells) for 2 replicate experiments on control HEK293 CPEs (C1, C2) and CPEs extracted from iAs-exposed HEK293T cells (S1, S2) when normalized to noRNA controls. For stress-control comparison, protein hits with a positive log-fold change in both replicates and a log- fold change ≧ 1 in at least one of the 2 replicates under stress conditions were selected and represented in maps. For actual log_2_ ratios, see supplementary Table 3.

(B) Heatmaps depicting protein associations with 5’ tsRNA-Glu^CUC/UUC^ (purified from Flp-In™ T-Rex™ 293-ecANG cells) for the same experiments as in (A) but normalized to scrambled RNA controls. For stress-control comparison, protein hits with a positive log-fold change in both replicates and a log- fold change ≧ 1 in at least one of the 2 replicates under stress conditions were selected and represented in maps. For actual log_2_ ratios, see supplementary Table 3.

**Supplementary Table legends**

**Supplementary Table 1**

Dynamic multiple reaction monitoring (MRM) parameters for the detection and quantification of modified nucleosides.

Abbreviations: MS1 Res, MS1 resolution; MS2 Res, MS2 resolution; Ret Time, retention time (with a one minute retention time window); SILIS, stable isotope-labelled internal standard (as described in^1^).

**Supplementary Table 2**

Results from biological triplicate experiments measuring tRNA- and 5’ tsRNA-Gly^GCC/CCC^ and tRNA- and 5’ tsRNA-Glu^CUC/UUC^ either after iAs exposure or ecANG expression. Absolute abundance of modified nucleosides per purified RNA (tRNA and 5’ tsRNAs derived from tRNA-Gly^GCC/CCC^ and tRNA-Glu^CUC/UUC^) was determined by isotope dilution mass spectrometry (as described in^1^).

**Supplementary Table 3**

List of proteins identified by mass spectrometry analysis of RNA affinity purified CPEs filtered for a minimum of three LFQ values over 12 LC-MS/MS experiments. Proteins enriched by endogenous RNA (endo) versus no RNA and scrambled RNA (controls) from stressed and steady-state conditions are listed separately based on the LFQ intensities ratios showing a positive log-fold change in both replicates and a log-fold change ≧ 1 in at least one of the 2 replicates.

**Materials and methods**

***Cell culture***

HEK293 and Flp-In™ T-Rex™ 293-ecANG cells were cultured in standard Dulbecco’s Modified Eagle’s Medium (DMEM, Sigma Aldrich) supplemented with penicillin (100 U/mL), streptomycin (100 μg/mL), 2 mM L-glutamine and 10% fetal bovine serum in a humidified incubator at 37°C and 5% CO_2_.

***Determining LD_50_ for iAs exposure***

HEK293T cells were cultured to 70% confluency, treated for 1 h with different concentrations of inorganic sodium arsenite (iAs) followed by washout of iAs and re-plating in fresh medium. After 24 h, cells were harvested by trypsinization, stained with Trypan-Blue and counted using a Neubauer-chamber.

***Stress experiments***

For oxidative stress, iAs was added at stated final concentrations to HEK293T cells at 70% confluency for 1 h, followed by washout and re-plating until harvest.

***Ectopic human ANG expression system***

A Flp-In™ T-Rex™ 293 cell line harboring an inducible expression cassette containing human Angiogenin-HA-FLAG (ecANG) was established after co-transfection of the ecANG destination vector and the pOG44 recombinase expression plasmid followed by clonal selection of cells transfected cells. This cell line was cultured under selection using 500 μg/mL Hygromycin B (stock: 100 mg/mL) and 75 μg/mL Blasticidin (stock: 10 mg/mL). At 70-80% confluency, ecANG expression was induced by adding doxycycline (Dox) to 1μg/mL. Cells were cultured in the continuous presence of Dox for up to 72 h.

***Determining cell viability during Dox-mediate ecANG expression***

Flp-In™ T-Rex™ 293-ecANG cells were cultured to 50% confluency, doxycycline was added to 1μg/mL and living cells were counted every day for three days using Trypan-Blue.

***Whole cell protein extraction***

For harvesting by scraping, cell culture media was removed from the cells and ice-cold 1x PBS was added. Cell pellets were homogenized in PD Buffer (20 mM Tris pH 7.4, 150 mM NaCl, 10 mM MgCl_2_, 10% (v/v) Glycerol, 0.2% (v/v) NP-40, 1x protease inhibitor cocktail, Roche) using a 26G needle (Sterican) followed by two centrifugations for 10 minutes at full speed at 4°C.

***Sub-cellular fractionation***

HEK293 cells were harvested into ice-cold 1x PBS. Cells were gently homogenized in CE-B (150 mM NaCl, 50 mM HEPES pH 7.4, 25 μg/ml digitonin, 1x protease inhibitor cocktail) for 10 minutes at 4°C under constant rotation followed by a centrifugation for 10 minutes at 2.000 x g and 4°C. The 2.000 x g supernatant was centrifuged an additional 10 minutes at full speed and 4°C, after which sediment was discarded and the remaining supernatant was considered as the soluble cytoplasmic fraction. The 2.000 x g pellet was washed in-ice cold 1x PBS followed by homogenization in ice-cold MOE-B (150 mM NaCl, 50 mM HEPES pH 7.4, 1% (v/v) NP-40, 1x protease inhibitor cocktail). Homogenates were incubated on ice for 30 minutes followed by centrifugation at 7.000 x g for 10 minutes to pellet nuclei. Nuclei were washed in ice-cold 1x PBS and homogenized in NE-B (20 mM Tris-HCl pH 8.0, 420 mM NaCl, 1.5 mM MgCl_2_, 0.2 mM EDTA, 1 mM PMSF, 25% (v/v) Glycerol, 1x protease inhibitor cocktail). Nuclei were extracted by rotation at 4°C overnight and the soluble nuclear protein fraction was collected by centrifugation for ten minutes at 7.000 x g at 4°C.

***Western blotting***

For whole protein lysates of HEK293 and Flp-In™ T-Rex™ 293-ecANG cells, cell pellets were homogenized in RIPA buffer (150 mM NaCl, 1% (v/v) NP-40, 0.1% (v/v) SDS, 50 mM Tris, pH 8.0, 0.5% (v/v) sodium deoxycholate, 1x protease inhibitor complex, Roche). Samples were incubated on ice for 10 minutes, followed by two centrifugations, 15 minutes each at 16.000 x g. 50 μg of total protein extracts were solubilized in SDS-sample buffer by boiling at 95°C for 5 minutes and analyzed by SDS-PAGE. Western blotting was performed with antibodies against human ANG (goat, R&D systems; 1:300), β-actin (rabbit, SIGMA; 1:1.000), eIF2α-P (rabbit, Abcam 32157; 1:500), HA (mouse 16B12, Covenance; 1:1.000), laminB (goat, Santa Cruz; 1:5.000).

***RNA extraction***

Collected cell pellets were re-suspended in 1x PBS and RNA was extracted using self-made Trizol solution (38% (v/v) phenol, 800 mM guanidine thiocyanate, 400 mM ammonium thiocyanate, 100 mM NaOAc, 5% (v/v) glycerol, 0.5% (w/v) N-lauroylsarcosine). Samples were incubated at room temperature for five minutes, followed by chloroform extraction and isopropanol precipitation. RNA pellets were washed in 75% ethanol and re-suspended in RNase-free water.

***Northern blotting***

RNA was separated on 12% Urea-PAGE in 0.5x TBE and transferred to Nylon membranes (Roche, GE Healthcare) using semi-dry blotting in 0.5x TBE for 30 minutes at 5 V=constant. Transferred RNA was immobilized by UV cross-linking (StrataLinker), followed by hybridization with ^32^P-end labeled oligonucleotides in blocking solution (5x SSC, 20 mM Na_2_HPO_4_ pH 7.4, 1% SDS, 1x Denhardt’s reagent) at 39°C overnight. After washing with 3x SSC, 5% (v/v) SDS for 15 minutes and 3x SSC, 5% (v/v) SDS for 10 minutes, membranes were exposed at room temperature to a magnetic screen and imaged using an Amersham Typhoon Biomolecular Imager (GE-Healthcare).

***Differential precipitation of small RNAs using Lithium chloride***

Harvested cell cultures were processed to separate high-molecular weight (HMW) from low-molecular weight (LMW) RNAs as described in^2^ but without pre-treatment steps. Instead total RNA was extracted using Trizol followed by LiCl precipitation of HMW RNAs overnight. The resulting soluble RNA content was precipitated with 1/10 volume of 3 M sodium acetate (pH 5.2) and 2.5 volume of pre-cooled absolute ethanol at −20°C overnight.

***tRNA and 5’ tsRNA isolation from HEK293 cells***

Total RNA was isolated from five 15 cm ⊘ cell culture plates exposed to 0.5 mM iAs for 1 h or from ecANG-expressing HEK293T cells after three days of Dox induction. In parallel, tRNAs from iAs-exposed cells were extracted. tsRNAs from iAs-exposed cells and ecANG-expressing cells were purified as follows. Total RNA was isolated from HEK293 cells using Trizol. Total RNAs extracted from five 15 cm ⊘ cell culture plates were re-suspended in 10 mL of IEX-buffer (20 mM Tris, 10 mM KCl, 1.5 mM MgCl_2_). For ion exchange chromatography, a HiTrap Q FF anion exchange chromatography column (1 mL, GE Healthcare) was used on an ÄKTA-FPLC (GE Healthcare) at 4°C. For elution, NaCl was used as eluent. Eluted fractions between 400-600 mM NaCl were collected and immediately precipitated using isopropanol at -20°C. RNA was re-suspended, supplemented with 10 mM MgCl_2_ and renatured by incubation at 75°C for three minutes. Afterwards the RNA was immediately placed on ice and added to 20 ml NHS-BB (30 mM HEPES KOH, 1.2 M NaCl, 10 mM MgCl_2_). For RNA affinity capture, 80 µg of a 5' amino-modified DNA oligonucleotide complementary to the target tRNA (IDT) was covalently coupled to a HiTrapTM NHS-activated HP column (1 mL, GE Healthcare). For isolation of full-length tRNAs, binding to the NHS-column was performed in a touch-down style. The small RNA pool was circulated for 90 minutes at 65°C and the temperature was gradually decreased over a three- h period until reaching 40°C and thereafter washed using NHS-WBA (2.5 mM HEPES KOH, 0.1 M NaCl, 10 mM MgCl_2_). For isolation of 5’ tsRNAs, the small RNA fraction in NHS-BB was circulated through the column for four h at 60°C and was afterwards washed using NHS-WBA. Bound RNAs were eluted by submerging the column in a water bath at 75°C in NHS elution buffer (0.5 mM HEPES-KOH, 1mM EDTA) followed by immediate precipitation in isopropanol at -20°C. To maintain column for reuse, it was washed with three volumes of NHS-SB (0.05 M Na_2_HPO_4_, 0.1% (w/v) NaN_3_, pH 7.0) and stored at 4°C. Precipitated RNA was re-suspended in water. Purified tRNA-Gly^GCC^ was further gel-purified (8% urea-PAGE in 0.5 x TBE) using RNA gel extraction buffer (0.3 M NaOAc, pH 5.2, 0.1% (v/v) SDS, 1 mM EDTA) and immediately precipitated in isopropanol at -20°C. Precipitated RNA was re-suspended in water.

***Isolation of ecANG by immuno-precipitation***

ecANG was isolated from cell culture supernatants of Dox-induced Flp-In™ T-Rex™ 293-ecANG cells. 72 h post-induction, supernatants were harvested and cleared of cells and debris by centrifugation. Dox-induced Flp-In™ T-Rex™ 293-ecANG cell supernatant (1 mL) was incubated with 50 µL anti-FLAG-M2 magnetic beads (50% suspension, SIGMA) for 1 h at room temperature, followed by three washing steps in PBS or buffers containing increasing concentration of NaCl. The amount of isolated ecANG was measured against defined masses of recombinant ANG using western blotting. Immuno-precipitated ecANG on beads was directly used for *in vitro* cleavage of tRNAs.

***Recombinant ANG- and ecANG-mediated tsRNA production***

For cleavage reactions using recombinant ANG (R&D Systems), 500 ng of total RNA or purified tRNA-Gly^GCC^ were melted for 5 minutes at 70 °C in H_2_O, put on ice for 5 minutes followed by addition of cleavage buffer (30 mM HEPES pH 6.8, 30 mM NaCl, 10 mM MgCl_2_). The reaction was incubated for 10 minutes at room temperature before adding either 100 ng of recombinant ANG to a final reaction volume of 15 µL or the reaction was added to ecANG on beads (from 1 milliliter cell culture supernatant) followed by incubation at 37°C for 2 h. The reaction was separated from magnetic beads and used for further analysis.

***DNAzyme-mediated tsRNA production***

500 ng of purified tRNA-Gly^GCC/CCC^ was incubated in a volume of 15 µl with a 10-fold excess of DNAzyme in 50 mM Tris-HCl (pH 7.5), 150 mM KCl, 10 mM MgCl_2_ (as described in ^48^). DNAzyme reactions were performed with 30 iterations of: denaturing step at 85°C for 30 seconds followed by incubation at 37°C for three minutes.

***Separation of residual tRNAs from tsRNA isolates***

To separate residual co-purified tRNAs from tsRNAs, precipitated RNAs were subjected to size exclusion chromatography. After re-suspending the affinity purified RNA in water in a total volume of 50 μL, the RNA was separated using an Advance Bio SEC 130A column (Agilent) in SEC buffer (20 mM Tris pH 7.4, 0.15 M NaCl) under a 0.75 mL/min flow rate. Fractions of 0.75 mL were collected and precipitated with isopropanol at - 20°C.

***Removal of residual tRNAs from tsRNA isolates using RNase H and gel purification***

To remove residual co-purified tRNAs from tsRNAs, precipitated RNAs were mixed with equimolar amounts of DNA oligonucleotides complementary to the 3’ half of the respective tRNA. The mix was heated to 80°C for three minutes, followed by reverse transcription using Superscript III (ThermoFisherScientific). In order to maintain tRNA-cDNA hetero-duplexes, final step of 85°C was omitted and the reaction was kept on ice. The reaction was supplemented with RNase H buffer and RNase H (10 U) and incubated at 37°C for 30 minutes for targeted degradation of tRNA. Exonuclease I (20 U, NEB) were added to digest cDNA and oligonucleotide sequences. Remaining tsRNAs were purified by acidic phenol-chloroform treatment and precipitation in isopropanol.

***Small RNA library preparation and cDNA sequencing***

100 ng of purified tsRNAs were treated with 0.5 U/µL T4 polynucleotide kinase (NEB) and 166 µM ATP (NEB) for 40 minutes at 37°C. After clean-up using acidic phenol-chloroform extraction followed by isopropanol precipitation, small RNA library preparation was performed using the NEBNext Multiplex Small RNA Library Prep Set for Illumina (Set 2) (NEB). cDNA libraries were sequenced on a HiSeq2000 platform in a spike-in format in paired-end 50 (PE50) mode.

***Small RNA sequencing analysis***

Small RNA reads were de-multiplexed, quality-controlled (fastqc) and trimmed (Trimmomatic) to remove adapters and barcodes, and to trim low-quality bases. Reads were mapped to the human genome (release hg19) using Bowtie2 and mapping parameters: -N 0 -L 22. Output alignment files were used to intersect with the coordinated of the predicted tRNA annotation in ENSEMBL and counted as total number of reads mapping to an annotated tRNA gene.

***LC-MS/MS analysis of RNA modifications***

About 400 ng of purified RNA was digested using a mixture of benzonase (2.5 U), bacterial alkaline phosphatase (10 U) and phosphodiesterase I (0.1 U) in a final reaction volume of 20 µL. The reaction mixture was supplemented with MgCl_2_ to a final concentration of 1 mM and Tris-HCl (pH 8.0) to a final concentration of 50 mM. Nucleobase deaminase inhibitor coformycin and tetrahydrouridine were added at a concentration of 10 μg/mL and 50 µg/ml, respectively, and butylated hydroxytoluene (an antioxidant) was added at a concentration of 0.5 mM (for further detail see^3^). The digestion was allowed to proceed for 2 h at 37°C and was stopped by filtering through a 10 kDa MWCO filter (AcroPrepTM Advance, 350 µl, OmegaTM 10K MWCO, Pall, Dreieich, Germany) at 3000 x g for 30 minutes. After addition of 10 µl pure water for salt dilution purposes, 18 µL of filtrate was mixed with 2 µL of internal standard (produced as recently described^4^). 10 µL of each sample was injected for LC-MS/MS analysis (corresponding to around 150 ng tRNA digest). Calibration solutions for absolute quantification were prepared as recently described^4^. For quantification, an Agilent 1290 Infinity II equipped with a DAD combined with an Agilent Technologies G6470A Triple Quad system and electro-spray ionization (ESI-MS, Agilent Jetstream) was used. Operating parameters were as follows: positive ion mode, skimmer voltage 15 V, Cell Accelerator Voltage 5 V, N_2_ gas temperature 230°C and N_2_ gas flow 6 L/min, sheath gas (N_2_) temperature 400°C with a flow of 12 L/min, Capillary Voltage of 2500 V, Nozzle Voltage of 0 V and the Nebulizer at 40 psi. The instrument was operated in dynamic MRM mode and the individual mass spectrometric parameters for the nucleosides are given in supplementary Table 1. The mobile phases were: (A) as 5 mM NH_4_OAc (≥ 99%, HiPerSolv CHROMANORM^®^, VWR) aqueous buffer, brought to pH = 5.6 with glacial acetic acid (≥ 99%, HiPerSolv CHROMANORM^®^, VWR) and (B) as pure acetonitrile (Roth, LC-MS grade, purity: 99.95%). A Synergi Fusion-RP column (Phenomenex^®^, Torrance, California, USA; Synergi^®^ 2.5 µm Fusion-RP 100Å, 150 x 2.0 mm) at 35°C and a flow rate of 0.35 mL/min was used. The gradient began with 100 % A for 1 minute, increased to 10% B by five minutes, and to 40 % B by 7 minutes. The column was flushed with 40% B for 1 minute and returned to starting conditions to 100% A by 8.5 minutes followed by re-equilibration at 100% A for 2.5 additional minutes.

***In vitro transcription of tsRNA sequences using hammerhead ribozymes***

Single-stranded DNA templates (IDT) encompassing a T7 promoter followed by hammerhead ribozyme and the specific 5’ tRNA isoacceptor or scrambled RNA sequences were PCR-amplified and afterwards *in vitro*-transcribed using 1.875 U/µL T7 Polymerase (NEB) in 1x T7-Buffer with 20 mM DTT, 18 mM spermidine, 3 µL murine RNase Inhibitors (40 U/µL NEB) and 125 µM NTP-mix (NEB). Transcribed and processed products were size selected on 8% urea-PAGE and subsequent extraction using RNA gel extraction buffer (0.3 M NaOAc (pH 5.4), 0.1% (v/v) SDS, 1 mM EDTA).

***Biotinylation of small RNAs***

5’ tsRNA-Glu^CUC^ was de-phosphorylated using 0.5 U/µg Fast-AP Thermosensitive Alkaline Phosphatase (ThermoFisher Scientific) in 1x Fast-AP Buffer for 12 minutes at 37°C followed by five minutes at 75°C. RNA was isolated using acid Phenol/Chloroform/Isoamylalcohol (P/C/I pH 4.5, ROTH) and precipitated using isopropanol. De-phosphorylated tsRNA or synthetic RNA (scrambled) were 5’-thiolated by incubation with 0.5 U/µL polynucleotide kinase (ThermoFisher Scientific) in the presence of 0.5 mM ATPγS (SIGMA) at 37°C overnight. EDTA was added to 1 mM final, PNK was inactivated by incubation at 75°C for five minutes and RNA was isolated using P/C/I, followed by precipitation in isopropanol. In each biotinylation reaction, 1 µg of small RNAs was mixed with 2 µg of HPDP-Biotin (ThermoFisher Scientific, 1 mg/ml in DMSO). The reaction was incubated for three h at room temperature protected from light and mixed every 15 minutes. To monitor successful biotinylation, an increase in absorbance at 343 nm, which reports on the accumulation of a reaction by-product, pyridine-2-thione as a proxy for the biotinylation efficiency, was measured using NanoDrop (ThermoFisher Scientific). Biotinylated small RNAs were separated from HPDP-biotin and pyridine-2-thione using spin columns (BioRad) in ultra-pure water.

***RNA affinity capture***

The RNA solution was supplemented to 10 mM MgCl_2_, denatured for three minutes at 75°C and cooled down to room temperature to re-nature the RNA. 450 µg CPE was pre-cleared by addition of 20 µl packed streptavidin-sepharose beads and incubation for 1 h at 4°C under rotation. 750 nanograms of biotinylated and re-folded RNA (measured by NanoDrop) was incubated for 30 minutes shaking at 1.500 rpm at room temperature with protein extract (500 µg of whole cell protein extract or 150 µg of CPE) and murine RNase Inhibitor (NEB) in the corresponding protein extraction buffer (without glycerol). Streptavidin beads were added to the RNA pull down reaction mixture, followed by incubation for 30 minutes shaking at 1.500 rpm at room temperature. Afterwards, streptavidin beads were washed three times with ice-cold protein extraction buffer (whole cell or cytoplasmic, without glycerol) for five minutes each while rotating at room temperature and twice with 1x PBS for five minutes rotating at room temperature. The beads were eluted by incubation for three minutes at 95°C with SDS-sample buffer.

***Proteolytic in-gel digest***

The eluates from 5’ tsRNA-Glu^CUC/UUC^ protein pull-down experiments were loaded on a SDS-PAGE and briefly stained with Coomassie Brilliant Blue. Stacked proteins were excised and gel pieces were washed, disulfide bridges were reduced with dithiothreitol and free thiols were alkylated with iodoacetamide. Proteins were digested overnight with trypsin (Promega) at 37°C. After digestion, peptides were extracted from the gel and cleaned-up on custom-made C18-stage tips^5^.

***Liquid chromatography – mass spectrometry (LC-MS/MS) for peptide identification***

Tryptic digests were separated on an Ultimate 3000 RSLC nano-flow chromatography system (ThermoFisher Scientific), using a pre-column for sample loading (PepMapAcclaim C18, 2 cm × 0.1 mm, 5 μm) and a C18 analytical column (PepMapAcclaim C18, 50 cm × 0.75 mm, 2 μm, Dionex-Thermo-Fisher Scientific), applying a linear gradient from 2- 35% solvent B (80% acetonitrile, 0.1% formic acid; solvent A 0.1% formic acid) at a flow rate of 230 nL/min over 60 minutes. Eluting peptides were analyzed on a Q-Exactive HFX Orbitrap mass spectrometer, equipped with a Proxeon nanospray source (all ThermoFisher Scientific). For the data-dependent mode survey scans were obtained in a mass range of 375–1.500 m/z with lock mass on, at a resolution of 60.000 at 200 m/z and an AGC target value of 3E6. The 8 most intense ions were selected with an isolation width of 1.6 Da, fragmented in the HCD cell at 28% collision energy and the spectra recorded at a target value of 1E5 and a resolution of 30.000. Peptides with a charge of +1 were excluded from fragmentation, the peptide match and exclude isotope features were enabled and selected precursors were dynamically excluded from repeated sampling for 15 seconds.

***Data analysis of protein identification***

Raw data were searched with MaxQuant software package 1.6.0.16^6^ against UniProt human reference database (proteome ID: UP000005640) and a custom contaminant database with tryptic specificity allowing two missed cleavages. Carbamidomethylation was set as fixed modification, oxidation of methionine and N-terminal protein acetylation as variable modifications. All other parameters were set to default. LFQ feature and match between runs were activated. Results were filtered at a protein and peptide false discovery rate of 1% at PSM and protein level. In Perseus 1.6.2.1, decoys and contaminants were filtered out, and the protein list reduced to entries with a minimum of one unique and razor peptide and a minimum of three LFQ values over 12 LC-MS/MS runs. LFQ values were log_2_-transformed, missing quantification values were replaced by a fixed value of 20. The protein intensity ratios (log_2_) for endo versus noRNA or scrambled RNA controls were calculated within replicates and plotted per condition. For stress-control comparison, protein hits with a positive log-fold change in both replicates and a log-fold change ≧1 in at least one of the two replicates under stress conditions were selected and represented in heatmaps.

***Immuno-fluorescence experiments***

Flp-In™ T-Rex™ 293-ecANG cells were plated onto poly-lysine-coated coverslips and Dox-mediated ecANG expression was induced for 48 h. Cells were briefly rinsed in ice-cold PBS, fixed with 4% PFA/1x PBS, washed with 1x PBS, blocked in blocking solution (3% bovine serum albumin, 0.1% Tx-100, 1x PBS) and stained with antibodies against human ANG in a wet chamber for 12 h. Cells were washed in blocking solution followed by incubation with secondary goat-anti-mouse Alexa488-coupled secondary antibodies (1:500) for 2 h at room temperature. Cells were washed in 1x PBS, exposed to Hoechst for DNA staining, embedded and imaged on a confocal microscope (Olympus FV3000).

***Determining tsRNA copy numbers in HEK293 cells***

A mass dilution of both purified 5’ tsRNAs (measured using NanoDrop, ThermoScientific) was northern-blotted using probes against the 5’ halves of tRNA-Gly^GCC^ and tRNA-Glu^CUC^ along with total cellular RNA extracted from HEK293 cells growing under steady state conditions and from cells exposed to iAs followed by immediate harvesting and RNA extraction. In parallel, the mass of RNA after total RNA extraction using Trizol from 1*10^6^ HEK293 cells was determined in triplicates using NanoDrop. Radiographic signals from the dilution series of 5’ tsRNAs were quantified using *ImageJ* and plotted as a standard curve using Excel. The signals collected from HEK293 cells were subtracted (stress minus steady state) to arrive at a mass of tsRNAs after iAs-exposure. The calculated mass of tsRNAs was used to arrive at the number of tsRNA molecules normalized to the mass of loaded total RNA. The number of tsRNA molecules per cell was calculated using the equation: moles ssRNA (mol) = mass of ssRNA (g)/(length of ssRNA (nt)* 321.47 g/mol) + 18.02 g/mol) where the length for a tRNA half was set to 35 nt (the anticodon triplet being nucleotides 34 to 36). RNA copy number was calculated as moles of ssRNA* 6.022*e^23^ molecules/mol.

***Primers and Oligonucleotides (5’ to 3’)***

*ecANG construct primers:*

ANG_Gateway_fwd: AD0004: GGGGACAAGTTTGTACAAAAAAGCAGGCTTCACCATGGTGATGGGCCTGGGCG

ANG_Gateway_rev: AD0005:

GGGGACCACTTTGTACAAGAAAGCTGGGTTCTAAGCGTAATCGGGAACATCG

*DNAzymes:*

V1: MATT_VIE361:

AACCCGGGCCTCCCGCGTGG_GGCTAGCTACAACGA_AGGCGAGAATTCTACCACTG

V2: MATT_VIE362:

TCGAACCCGGGCCTCCCGCG_GGCTAGCTACAACGA_GGCAGGCGAGAATTCTACCA

*in vitro transcription (IVT)/ribozyme single-stranded DNA template:*

scrambled small RNA: AD114: GGTGACTGGAGTTCAGACGTGTGCTCTTCCGATCTGACGGTACCGGGTACCGTTTCGTCCTCACGGACTCATCAGAGATCGGAATCTCCCTATAGTGAGTCGTATTA

*PCR primers for making double-stranded IVT/ribozyme templates:*

scrambled small RNA_fwd: AD115: GGTGACTGGAGTTCAGACGTG

universal_rev: MATT_VIE174: CGCGCGAAGCTTAATACGACTCACTATA

*Northern blotting probes:*

5’ tRNA-Gly^GCC^: AD0006: TCTACCACTGAACCACCAAT

3’ tRNA-Gly^GCC^: AD0007: TGGTGCATTGGCCGGG

5’ tRNA-Glu^CUC^: AD0008: GAATCCTAACCACTAGACCAC

*RNA affinity capture probes:*

tRNA-Glu^CUC^: AD0022: 5'-Amino-Modifier C6-CGCCGAATCCTAACCACTAGACCACCA

tRNA-Gly^GCC^: AD0023: 5'-Amino-Modifier C6-AGGCGAGAATTCTACCACTGAACCACC

**Supplementary References**

1. Heiss M, Reichle VF, Kellner S. Observing the fate of tRNA and its modifications by nucleic acid isotope labeling mass spectrometry: NAIL-MS. RNA Biol 2017; 14:1260–8.

2. Peng J, Xia Z, Chen L, Shi M, Pu J, Guo J, Fan Z. Rapid and efficient isolation of high-quality small RNAs from recalcitrant plant species rich in polyphenols and polysaccharides. PLoS ONE 2014; 9: e95687–.

3. Cai WM, Chionh YH, Hia F, Gu C, Kellner S, McBee ME, Ng CS, Pang YLJ, Prestwich EG, Lim KS, et al. A Platform for Discovery and Quantification of Modified Ribonucleosides in RNA: Application to Stress-Induced Reprogramming of tRNA Modifications. Meth Enzymol 2015; 560:29–71.

4. Borland K, Diesend J, Ito-Kureha T, Heissmeyer V, Hammann C, Buck AH, Michalakis S, Kellner S. Production and Application of Stable Isotope-Labeled Internal Standards for RNA Modification Analysis. Genes 2019; 10:26.

5. Rappsilber J, Mann M, Ishihama Y. Protocol for micro-purification, enrichment, pre-fractionation and storage of peptides for proteomics using StageTips. Nature protocols 2007; 2:1896–906.

6. Cox J, Mann M. MaxQuant enables high peptide identification rates, individualized p.p.b.-range mass accuracies and proteome-wide protein quantification. Nat Biotechnol 2008; 26:1367–72.
