# Supplementary figures and images for: Production and purification of endogenously modified tRNA-derived small RNAs
Source: RNA Biol. 2020 Mar 5;17(8):1104–15. doi: 10.1080/15476286.2020.1733798 (PMC7549616; doi:10.1080/15476286.2020.1733798)

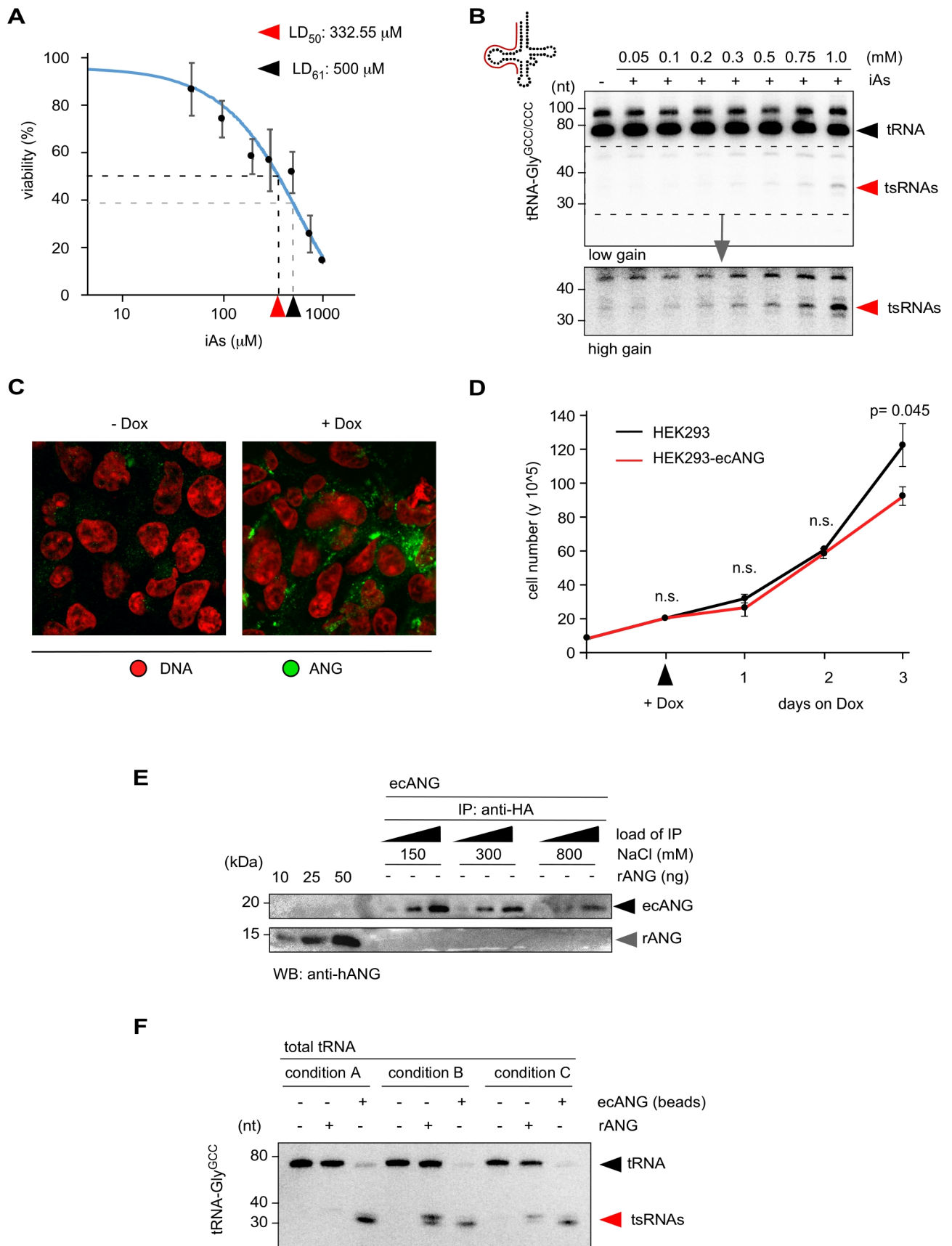

Supplement: Supplemental Material [file KRNB_A_1733798_SM0715.zip › Supplementary information/SFigure_1R.pdf]

**A**

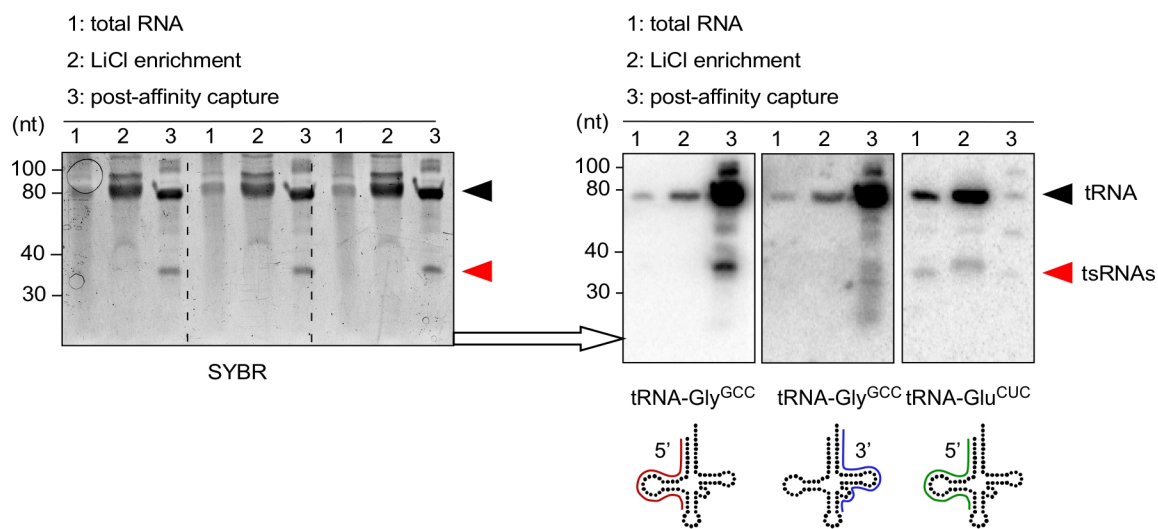

**B**

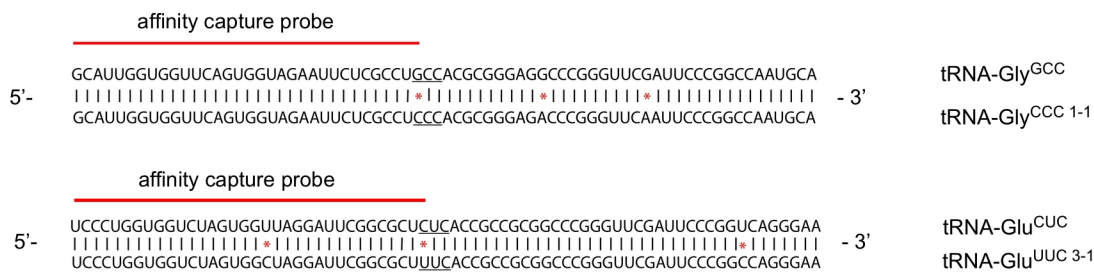

Supplement: Supplemental Material [file KRNB_A_1733798_SM0715.zip › Supplementary information/SFigure_2R.pdf]

A

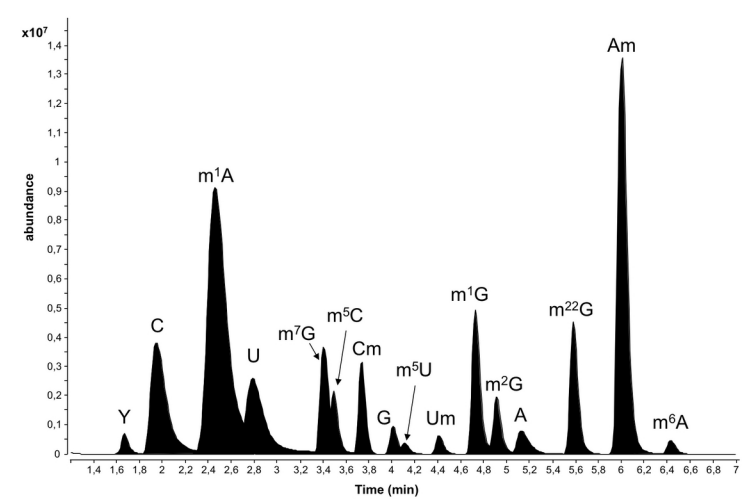

B

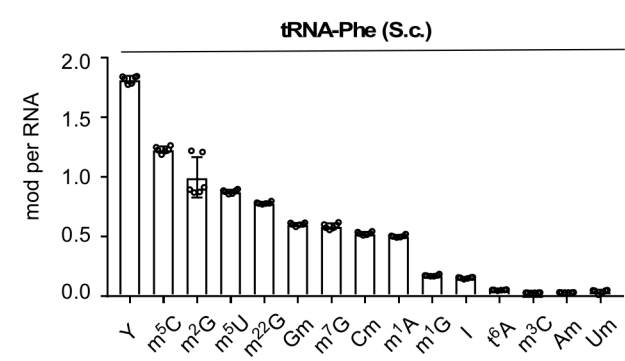

Supplement: Supplemental Material [file KRNB_A_1733798_SM0715.zip › Supplementary information/SFigure_3R.pdf]

A

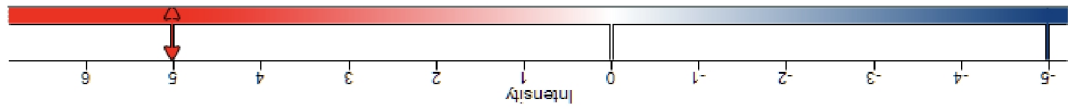

B

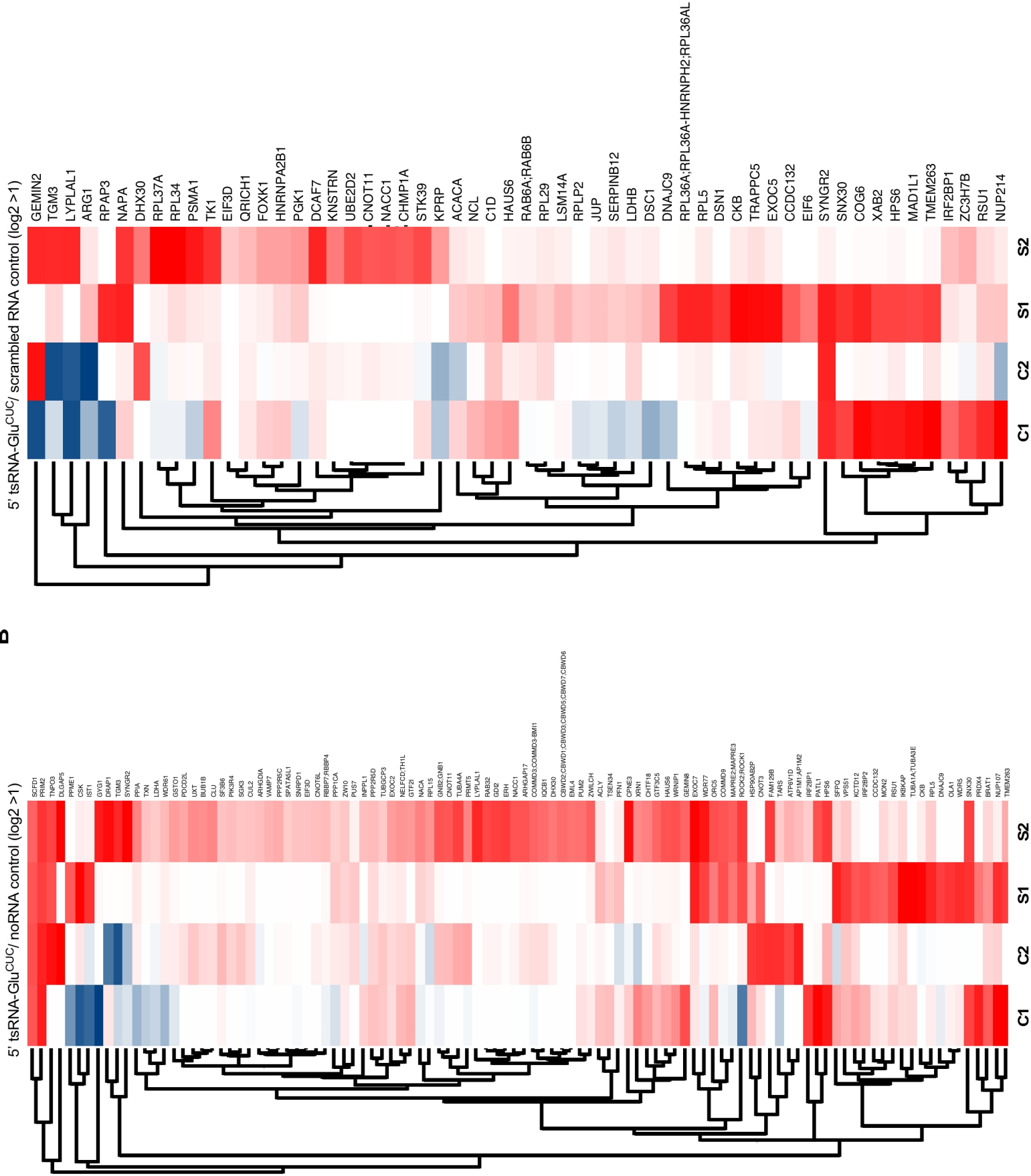

Supplement: Supplemental Material [file KRNB_A_1733798_SM0715.zip › Supplementary information/SFigure_4R.pdf]
